# Study protocol for the development of a digital menstrual cycle diary for routine mental health and gynecological care: A human-centered design approach

**DOI:** 10.1371/journal.pone.0342586

**Published:** 2026-03-09

**Authors:** Michèle Schmitter, Astrid Cantineau, Marije aan het Rot, Annemiek Lely, Tom Verhage, Michelle N. Servaas, Harriëtte Riese

**Affiliations:** 1 Department of Psychiatry, University Medical Center Groningen, University of Groningen, Groningen, The Netherlands; 2 Depression Expertise Centre, Pro Persona Mental Health Care, Nijmegen, The Netherlands; 3 Department of Obstetrics and Gynecology, University Medical Center Groningen, University of Groningen, Groningen, The Netherlands; 4 Department of Psychology (Clinical), University of Groningen, Groningen, The Netherlands; 5 Dutch Brain Foundation, The Hague, The Netherlands; PLOS: Public Library of Science, UNITED KINGDOM OF GREAT BRITAIN AND NORTHERN IRELAND

## Abstract

**Background:**

Premenstrual disorders, including premenstrual syndrome, premenstrual dysphoric disorder, and premenstrual exacerbation of psychiatric disorders affect a significant portion of reproductive-age females. Accurate diagnosis and tailored treatment are often constrained by the limitations of traditional paper-based symptom diaries. These diaries lack flexibility for personalized symptom tracking and fail to capture treatment-relevant factors such as lifestyle and social events. A digital, adaptable symptom diary is therefore needed. In this paper, we present the protocol for the CycleWise study. This study is a multi-method study aimed at developing a digital menstrual cycle diary within PETRA (PErsonalized Treatment by Real-time Assessment), an ESM-based tool previously designed to support treatment in routine mental health care.

**Methods and analysis:**

Following a human-centered design approach and the Centre for eHealth and Wellbeing Research roadmap, patients and clinicians will be involved throughout all phases to ensure the tool meets their needs. In the contextual inquiry phase, we will identify stakeholders and analyze current practices. The value specification phase will focus on assessing stakeholder needs through two focus groups and translating them into functional requirements. A user experience designer will then develop a prototype in the design phase, refining it iteratively based on stakeholder feedback. Implementation strategies will be formulated in the operationalization phase. Finally, uptake, impact and working mechanisms will be evaluated through qualitative interviews and quantitative measures.

## Introduction

Many reproductive-age females report physical or emotional symptoms during the luteal phase of their menstrual cycle [1], with over 200 symptoms documented [2]. Common symptoms include breast tenderness, bloating, headaches, food cravings, tiredness, mood swings, depression, anxiety, and anger. Although these symptoms are typically mild, a subset of females experience them with sufficient severity to impair daily functioning [3,4]. Approximately 20–30% meet diagnostic criteria for premenstrual syndrome (PMS) [5,6], marked by one or more emotional or physical symptoms [7]. A smaller proportion (2–8%) meet criteria for premenstrual dysphoric disorder (PMDD) [8], a psychiatric disorder classified in the Diagnostic and Statistical Manual of Mental Disorders (DSM-5) [9]. PMDD requires the presence of at least five DSM‑listed symptoms during the luteal phase, including core emotional symptoms, that cause significant distress or functional impairment [9]. A diagnosis of PMS or PMDD additionally requires a symptom-free period of at least one week following menstruation. When symptoms persist into the follicular phase, albeit at reduced intensity, this pattern may indicate premenstrual exacerbation (PME) of an underlying psychiatric disorder rather than PMS or PMDD [10]. PME has been documented across a wide range of psychiatric disorders, including anxiety [11,12], depressive [11,13], bipolar [11,13], psychotic [11,14], personality [15], eating [16,17], substance use [11,18], and attention-deficit/hyperactivity disorder (ADHD) [19], with prevalence estimates ranging from 20% to 60% [11,13]. In this paper, we define premenstrual disorders (PMDs) as encompassing PMS, PMDD, and PME. PMDs substantially impair daily functioning, contribute to productivity loss and increased healthcare utilization [6,20], and heighten the risk of suicidal ideation [21,22]. In the case of PME, symptom cyclicity may also shorten the time to relapse following otherwise effective treatment [23]. Given these significant clinical and functional consequences, routine assessment of menstrualcycle–related symptom patterns is urgently needed to support accurate diagnosis and effective treatment of PMDs [24,25].

According to guidelines [7,9,10], accurate diagnosis of a PMD requires prospective symptom tracking across at least two menstrual cycles. Several paper-based symptom diaries exist, such as the Daily Record of Severity of Problems (DRSP) [26], widely used in clinical practice to diagnose PMDD according to DSM-5 criteria, and the PMS calendar test, which tracks the ten most common premenstrual symptoms [7,27]. However, these diaries have several limitations. First, their emphasis on a fixed list of symptoms—predominantly DSM-defined PMDD and somatic symptoms—does not fully capture the heterogeneity of symptoms experienced by individuals with PME or PMS [2]. Second, they also omit important treatment-relevant information such as lifestyle behaviors [28–31] or daily life events and response patterns [32], which can support tailored interventions. Third, patients may forget to carry them, miss entries, or retrospectively report symptoms, introducing inaccuracies [33]. Finally, paper diaries can be lost or accessed by others, raising privacy concerns.

Smartphone-based diaries offer flexible, personalized symptom tracking, support compliance, and enable more accurate daily monitoring [34]. Smartphones have become an indispensable part of modern life, with mobile cellular subscriptions worldwide estimated at 110 per 100 individuals [35], and menstrual cycle apps are widely used to track ovulation and menstruation [36]. The CycleWise study aims to overcome the limitations of traditional paper-based diaries by developing a smartphone-based digital menstrual cycle diary. This tool is designed to support routine mental health and gynecological care, addressing the urgent need to integrate menstrual cycle information into standard practice [24,25].

To ensure the digital menstrual cycle diary meets end-user needs, its development follows the Centre for eHealth and Wellbeing Research (CeHRes) roadmap for eHealth development [37]. This iterative framework consists of five interrelated phases, which are; 1) contextual inquiry, 2) value specification, 3) design, 4) operationalization, and 5) summative evaluation. End-users are involved throughout all phases [37]. The contextual inquiry phase involves identifying key stakeholders and analyzing the current situation to determine areas for improvement [37]. In the value specification phase, the needs and expectations of end-users are translated into specific eHealth requirements. The design phase focuses on developing prototypes and iteratively refining them based on end-user feedback. In the operationalization phase, implementation strategies are developed. Finally, the summative evaluation phase assesses the uptake and impact of the eHealth application [37]. Formative evaluation occurs throughout all phases as an ongoing, iterative process that keeps the technology and development activities aligned with stakeholder perspectives and prior phase outcomes.

This protocol paper details the CycleWise study procedures for each CeHRes phase in developing the digital menstrual cycle diary for routine mental health and gynecological care. By co-designing the diary with end-users, we aim to develop a diary that fits their needs, contributes to diagnostic accuracy, supports tailored treatment, and strengthens the therapeutic alliance between patients and clinicians.

## Materials and methods

Participation in this study involves minimal risk, as patients primarily provide input on the design of a digital menstrual cycle diary and use the diary in routine care. Patient recruitment will take place from April 30, 2025 to December 1, 2026, with data collection concluding in March 2027 and results expected by September 2027. Standard data protection measures will be applied, and ethical approval has been obtained from the Central Ethical Review Committee of the University Medical Center Groningen (UMCG; Registration number: 21338). All participants will provide informed consent prior to participation. The study was designed and will be reported in accordance with the COnsolidated Criteria for Reporting Qualitative REsearch checklist (COREQ) checklist [38].

### Data management

Data to be managed include qualitative data (i.e., focus groups and interviews), survey data, logistic data, personal data and diary data. Focus group data will be anonymized, with only general information on participants’ roles, professions, or diagnoses reported. Individual focus group contributions will not be linked to demographic or survey data. Screening information, interviews and quantitative survey responses, however, will be linked using a unique participant identifier stored in a secure key file. Throughout the study, audio files and transcripts of qualitative data, as well as personal data, will be stored separately on the secured UMCG drive. Surveys will be administered via RoQua [38], a secure web application for outcome monitoring in health care and research. Logistic data will be captured in REDCap (Research Electronic Data Capture) [39], a secure platform for managing research databases. REDCap data are automatically backed up daily on the internal servers. Logistic data include all information related to the execution of research activities, captured through digital checklists at each procedural step. Upon study closure, REDCap will be frozen following verification and resolution of all queries. All raw data files, as well as qualitative and personal data, will be stored in password-protected folders, locked for editing, and labeled with the save date.

The final menstrual cycle diary will be embedded within an Experience Sampling Method (ESM)-based tool named PErsonalized Treatment by Real-time Assessment (PETRA), which was previously developed to support treatment of patients in mental health care [40]. ESM is a scientific method commonly used for self-monitoring in mental health care [41]. PETRA is a scientific, web-based platform that is integrated into the Electronic Health Record system in RoQua across eight major Dutch mental health care institutions and is also accessible to other UMCG departments, including gynecology. Diary data are therefore stored directly in a patient’s Electronic Health Record. In the PETRA platform, informed consent for the use of diary data in research is embedded in the user interface and legally secured through the PETRA consortium agreement, enabling future ESM studies. PETRA was co-designed with psychiatric clinicians and patients [40], and is available in Dutch and English [42]. It is a flexible tool used in routine mental health care to support various goals, such as monitoring treatment progress and helping clinicians better understand patients’ momentary experiences to inform diagnosis and personalized treatment. In short: PETRA consists of four key components: 1) a goal-setting decision aid, 2) a repository of diary items, 3) an SMS-based diary delivery system, and 4) a feedback module. In line with patient and clinician preferences, PETRA allows personalization of the digital diary content, schedule, and duration of monitoring. Its ESM-based feedback module includes descriptive graphs and summary statistics and aims to support data-driven discussions and shared decision-making during diagnostic assessments and treatment.

### CycleWise study design

The menstrual cycle diary development follows a human-centered, iterative design approach guided by the CeHRes roadmap and employs a multimethod strategy [37]. Because this study is not a clinical trial, trial registration is not applicable.

### Study procedures per CeHRes roadmap phase

**Fig 1:**
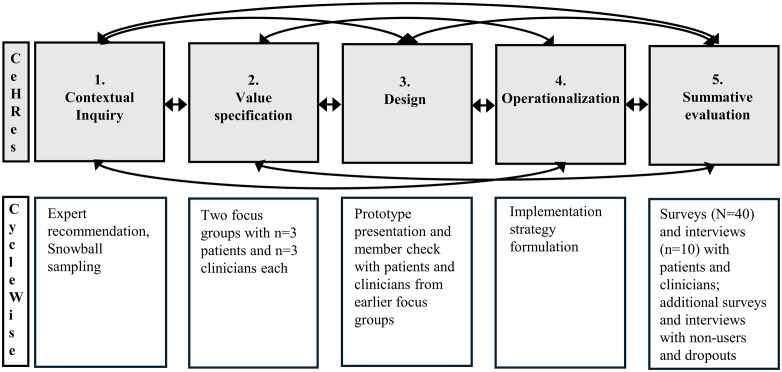
Overview of the CeHRes Roadmap phases and their application in this study. The upper layer outlines the CeHRes roadmap phases, while the lower layer illustrates how these phases are applied in the CycleWise study.

Below, we first outline how each phase of the CeHRes roadmap is implemented in this study, along with the specific setting and procedures for each of the five phases before moving to participant criteria. Fig 1 presents an overview of the research activities associated with each phase.

#### Phase 1: Contextual inquiry.

The contextual inquiry phase aims to map the relevant stakeholders and analyze current practices to identify opportunities for improvement. Stakeholders include clinicians from mental health and gynecological care institutions (e.g., gynecologists, psychologists, psychiatrists, nurses), as well as patients with a (suspected) PMD. To identify relevant stakeholders for the co-design process, we will use expert recommendations and snowball sampling, whereby identified stakeholders refer others within their network [37]. The multidisciplinary project group includes clinical psychology researchers, a health psychologist, a gynecologist, a person with lived experience, and a user experience (UX) designer, reflecting current best-practice recommendations for eHealth development [40]. The project group will initiate stakeholder identification. Once clinicians are engaged, they may refer patients with PMDs. Additionally, we will collaborate with patient organizations, including the Dutch Brain Association, the internal UMCG psychiatry patient panel, and the Dutch PMDD organization to recruit individuals with a (suspected) PMD diagnosis. If needed, we will also use social media sites for recruitment.

Potential participants receive a flyer from their treating clinician or via the patient organizations involved. The flyer contains a QR-code which links to a short survey via which patients can provide consent to be contacted and contact information. A project group member will then email detailed study information and the study consent form. After a minimum of 48 hours, researchers will call patients to address any questions, assess eligibility, and confirm participation. Informed consent will be collected digitally or, if needed, using a printed form.

#### Phase 2: Value specification.

The aim of the value specification phase is to identify user needs and translate them into functional requirements for the digital menstrual cycle diary. To achieve this, we will conduct two mixed focus groups with patients and clinicians. A maximum variation sampling strategy [39] will be applied to ensure that participants represent diverse experiences and backgrounds.

All participants will first provide demographic information. Additionally, patients will report basic clinical and menstrual cycle details during a screening interview (i.e., menstrual status, contraceptive use, date of last menstruation, cycle regularity, average cycle duration, help-seeking for a suspected PMD, the experience of symptom fluctuations across the cycle and tracking methods used; see also supporting information Table A in S1 File). Clinicians will be selected based on age, gender identity, and profession, while patients will be selected based on age, education level, referral pathways (e.g., past or current referrals to gynecology or psychiatry), the (suspected) type of PMD diagnosis, and prior experience with paper-based or digital menstrual cycle diaries.

The focus groups will be conducted in person at the UMCG and led by a female postdoctoral researcher (MS1), a male UX designer (TV), and a female with lived experience (AL). Each session will last approximately two hours. We will follow a semi-structured topic guide including questions like ‘What do you want to learn from monitoring the menstrual cycle and related symptoms?’, ‘What would make the digital menstrual cycle diary better or worse than the paper diary?’, ‘What should the menstrual cycle diary monitor?’ and ‘What is most important for you to learn after completing the menstrual cycle diary?’. The full topic guide is provided in the supporting information (Table B in S1 File). Participants will be encouraged to ask each other questions and discuss their perspectives. TV and AL will take notes during the discussions, which will be used by the research team to create a session summary. After each session, participants will be invited to review the summary in a member check and provide any feedback by email to confirm the accuracy of the interpretations [40].

If key stakeholders, such as the lead gynecologist or psychiatrist involved in female-sensitive care at the UMCG, are unable to attend the scheduled focus groups, they will receive a summary of the discussion and be invited to share their input afterwards. Even if they are not directly involved in the sessions, their expert feedback remains valuable to the overall process, as they will be among the primary end-users.

#### Phase 3: Design.

Based on the value specification phase, the UX designer will develop a prototype of the digital menstrual cycle diary and its feedback module. The prototype will be presented to the same stakeholders from the value specification phase, and their feedback will inform further refinement of the diary. This meeting will also be conducted in person at the UMCG and led by the same female postdoctoral researcher (MS1), male UX designer (TV), and female with lived experience (AL).

The aim of the session will be to assess whether the prototype meets the needs identified in the value specification phase. A clinician and patient will use the prototype digital menstrual cycle diary to set up the diary for the patient and review the feedback. Participants will be asked to think aloud while navigating the prototype, sharing their impressions, noting what works well, what is unclear, what is missing, and providing feedback on usability. The other participants will observe and provide additional feedback. MS1 and AL will take notes during the session, and participants will be invited to review the summary of main feedback points in a member check to confirm the accuracy of the interpretations.

To ensure the diary meets the intended B1 language level, we will collaborate with the Jasmijn Groningen Foundation, a center supporting females with a migration background. During an in-person session, one participant will assess the prototype for linguistic accessibility. The postdoctoral researcher (MS1) and UX designer (TV) will be present to propose clearer, more accessible wording if needed, which the participant will review and confirm. Participants in both the value specification and design phase will receive a gift card valued at €25 per hour of participation. An external software development firm, Researchable B.V., will program the final prototype, which will then be integrated into PETRA via RoQua.

#### Phase 4: Operationalization.

The goal of the operationalization phase is to execute the practical rollout of the menstrual cycle diary, ensuring its introduction, dissemination, and integration into routine practice. In the preceding phases, we will briefly explore factors that may influence implementation, which will subsequently inform the development of targeted strategies in the operationalization phase. Although the topic guides from earlier phases do not include explicit questions about implementation barriers, we will remain attentive to any spontaneously mentioned considerations, such as clinicians’ knowledge, workflow integration, or support needs. Early discussions with clinical teams during recruitment are also expected to provide insight into how the menstrual cycle diary can be incorporated into routine practice. These insights, together with the expertise of the project group and guidance from PETRA implementation specialists, will inform strategy development [41]. Strategy selection will be guided by an established taxonomy, such as the Expert Recommendations for Implementing Change (ERIC) framework [42].

#### Phase 5: Summative evaluation.

The goal of the summative evaluation is to determine the digital menstrual cycle diary’s impact, uptake, and working mechanisms, assessed through interviews and evaluation surveys following diary use. Patients and their treating clinicians with PETRA access via the Electronic Health Record system will use the diary as part of routine care: clinicians initiate and tailor the diary with patients and review the PETRA‑generated feedback during regular consultations, similar to existing paper‑based diaries. Participation in the summative evaluation (interview and end-of-study evaluation survey) is optional, and patients and clinicians may participate either jointly or independently.

Clinicians will introduce the evaluation study to their patients and provide a flyer with a QR-code linking to the contact form. After receiving study information and consenting to be contacted, patients undergo the same screening procedure described in the contextual inquiry phase, followed by an informed consent procedure identical to that used in the focus groups. Consenting patients complete a one-time assessment (PSST [43]; OQ-45 [44], SCL-90-R [45]; see Materials section) to characterize the sample. Surveys are administered at the start of menstruation to facilitate recall of the premenstrual week.

To assess usability, impact, and working mechanisms, patients, clinicians, and—where relevant—other staff involved in diary setup will participate in semi-structured interviews (see interview guide, Table C and D in S1 File) and complete an end-of-study evaluation survey. The survey includes the TWente Engagement with EHealth Technologies Scale (TWEETS) for patients [46] or the adapted Professionals Engagement with E-Health Technologies Scale (PEEHTS) for professionals [47], along with additional items assessing the diary’s impact and the feedback module. These additional items will be iteratively developed by the project group after the design phase, informed by identified stakeholder needs and the key evaluation components outlined in the CeHRes Roadmap [37]. Patients will receive a €15 gift card for completing the one-time assessment and end-of study evaluation survey, and another €15 gift card for participating in the interview.

Interviews will be conducted by the postdoctoral researcher (MS1) at the UMCG or online via TEAMS, with each session expected to last approximately 30 minutes. Participants will have the opportunity to review a summary of their interview in a member check and provide feedback via email to confirm the accuracy of the interpretations. To capture diverse perspectives, we will also include patients who discontinue diary use and systematically investigate reasons for discontinuation through the evaluation survey. Patient compliance with the digital diary (i.e., the number of completed diary entries) will provide an additional quantitative measure of usability. Finally, a confidential survey (Table E in S1 File) will be administered to clinicians who never used the menstrual cycle diary, aiming to identify potential barriers to adoption. The results from this phase will inform improvements to the menstrual cycle diary.

### Participants

For participation in the CeHRes roadmap–defined value specification phase, patients must be 18 years or older and have a past, current, or suspected PMD diagnosis. Eligibility will be assessed during the screening interview (Table A in S1 File; questions 1, 2, or 12). Clinicians will be eligible if they provide care for patients diagnosed with a PMD, including professionals from routine gynecology or psychiatric care services. Exclusion criteria for both patients and clinicians include insufficient proficiency in Dutch.

In the evaluation phase, eligible patients will also be 18 years or older and have a past, current or suspected PMD diagnosis. Additionally, these patients must be referred for psychiatric or gynecological treatment at the UMCG or another mental health care institution with access to PETRA. Additional criteria include having a regular menstrual cycle with a cycle length between 21–35 days and the ability to provide informed consent. Exclusion criteria are acute medical conditions hindering participation, insufficient proficiency in Dutch, or unwillingness or inability to use a smartphone. Clinician eligibility criteria will be identical to those in the value specification phase.

### Sample size

In the value specification phase, two focus groups (each n = 3 patients and n = 3 clinicians) will be conducted to identify user needs. The second group will help verify and refine the needs identified in the first. A member check, involving the same patients and clinicians from the previous groups, will be conducted to evaluate the prototype in the design phase. The sample size is based on prior research indicating that two to three focus groups with six to eight participants typically cover at least 80% of thematic content [48].

In the evaluation phase, the primary assessment method is the semi‑structured interview. Sample size will be guided by the principle of data saturation; we anticipate reaching thematic saturation with approximately 10 patient and 10 clinician interviews [49], but additional participants will be recruited if new themes continue to emerge. The quantitative evaluation survey will be distributed to approximately 20 patients and 20 clinicians to descriptively assess user experiences and to triangulate and complement the qualitative findings.

### Material

All interview and focus group guides, along with the survey for participants who never used the menstrual cycle diary, are provided in the supporting information. These materials were iteratively reviewed by the project group to reach consensus on content and items. To describe the sample of the evaluation phase, patients will answer a one-time assessment, consisting of the PSST, OQ-45 and SCL-90-r.

The Dutch version of the PSST will be used to retrospectively assess PMS and PMDD diagnoses [43]. The PSST consists of two main sections with a total of 19 items (±5 minutes). The first section evaluates physical and psychological symptoms (14 items), while the second section (5 items), assesses the impact of these symptoms on the patients’ daily life. Each item is rated on a 4-point Likert scale, ranging from ‘not at all’ (0) to ‘severe’ (3). According to the PSST, PMDD is diagnosed when at least one core emotional symptom is severe, four or more additional symptoms are moderate to severe, and at least one domain of functioning is severely impaired. Moderate to severe PMS is diagnosed when at least one core emotional symptom and four or more additional symptoms are moderate to severe, with at least one moderate to severe functional impairment. Cronbach’s alphas for the first and second sections have previously been reported as 0.96 and 0.91, respectively [50], indicating excellent internal consistency.

The Dutch version of the OQ-45 will evaluate psychological functioning [44]. The OQ consists of 45 items (±10 minutes) that are scored on a 5-point Likert scale, ranging from ‘never’ (1) to ‘almost always’ (5). Three subscales measure psychological functioning in different domains: symptom distress (25 items), interpersonal relations (11 items) and social role performance (9 items). Cronbach’s alphas for the subscales and total score have previously been reported as 0.91, 0.80, 0.69 and 0.93, respectively in clinical samples [51], indicating acceptable to excellent internal consistency.

The Dutch version of the SCL-90-r will assess the severity of physical and psychiatric symptoms (45). The SCL-90-r contains 90 items (±20 minutes), each scored on a 5-point Likert scale ranging from ‘not at all’ (0) to ‘very much’ (4). Items are distributed across eight symptom dimensions (somatization; 12 items, obsessive–compulsive; 10 items, interpersonal sensitivity; 9 items, depression; 13 items, anxiety; 10 items, hostility; 6 items, phobic anxiety; 7 items, paranoid ideation; 6 items, psychoticism; 10 items) and three global indices (Global Severity Index, Positive Symptom Distress Index, Positive Symptom Total). Cronbach’s alphas have previously been reported to range between 0.69 and 0.87 for the subscales [52], indicating acceptable to good internal consistency.

As part of the end-of-study evaluation survey, we will assess patient engagement using the TWEETS [46] and professional engagement using the PEEHTS [47]. Both 9-item scales evaluate behavioral, cognitive, and affective engagement (±5 minutes), with responses scored on a 5-point Likert scale ranging from ‘strongly disagree’ (1) to ‘strongly agree (5).’ Cronbach’s alpha has been reported as 0.86 for the TWEETS [46] and 0.89 for the PEEHTS [47], indicating good internal consistency.

### Analytical approach

Qualitative data will be analyzed using the Qualitative Analysis Guide of Leuven (QUAGOL), a systematic framework for qualitative research [53]. Following QUAGOL, two researchers will first familiarize themselves with the focus group and interview transcripts through repeated readings and the creation of narrative summaries, which will then guide subsequent inductive coding and thematic analysis using ATLAS.ti software (version 23).

For both focus groups and interviews, two independent raters will code the transcripts and reconcile differences through consensus discussions [53]. For the interviews, an initial code list will be developed based on the first two transcripts and iteratively refined as new data emerge. Resulting codes from both data sources will be organized into clusters to inform the development of broader themes, which will be reviewed and validated in team meetings to ensure consistent interpretation. Quantitative data from the evaluation survey will be analyzed using descriptive statistics.

## Discussion

The CycleWise study aims to develop a digital menstrual cycle diary tailored for routine mental health and gynecological care, facilitating personalized symptom tracking across the menstrual cycle in patients with premenstrual disorders. Guided by a human-centered design approach and the CeHRes roadmap [37], the development process emphasizes continuous end-user involvement to ensure the tool aligns with the needs of both patients and clinicians. Addressing a critical gap in clinical practice, the tool will provide a flexible and user-friendly alternative to traditional paper-based diaries. The digital menstrual cycle diary will be embedded in an already developed and implemented digital diary system. This approach supports high-frequency longitudinal symptom tracking and the integration of treatment-relevant information, which are essential for understanding symptom dynamics and optimizing personalized care [54,55]. This tool has the potential to support improvement of diagnostic accuracy, treatment personalization, and enhance the therapeutic alliance, with the ultimate goal of improving care for patients diagnosed with premenstrual disorders.

### Strengths and limitations

This study has several notable strengths. First, it helps filling a critical gap by developing a digital tool to support the diagnosis and personalized treatment of premenstrual disorders, which are often under-recognized in mental health care and research [24,25]. Multidisciplinary collaboration ensures the diary’s relevance across specialties involved in the routine care of patients with premenstrual disorders. Engaging stakeholders throughout all phases of tool development increases the likelihood of successful adoption and sustained use in clinical practice [37]. The multi-phase, iterative study design allows for continuous refinement based on end-user feedback, while integration into existing workflows supports seamless implementation. Testing in routine clinical settings with both patients and clinicians ensures real-world applicability. The mixed-method approach provides a comprehensive evaluation of usability and impact.

However, the study is not devoid of limitations. This study focuses on short-term (i.e., two menstrual cycles) usability and acceptability, without follow-up on long-term adoption or clinical outcomes. Selection bias may also be a concern, as patients who choose to participate in focus groups may be more motivated or comfortable discussing menstrual health, while others may be deterred by ongoing stigma surrounding the menstrual cycle [56], potentially limiting the diversity of perspectives captured. Moreover, the generalizability of the findings is limited. PETRA is currently available in only eight Dutch mental health care institutions and for research purposes on demand, which may restrict the diversity of clinical settings where the digital menstrual cycle diary can be implemented. However, PETRA is technically ready and adaptable for broader implementation. The PETRA team is open to collaborations in both (mental) health care and research settings, supporting future expansion and evaluation across more diverse contexts.

Despite these limitations, this study represents an important step toward addressing the unmet need for a flexible digital menstrual cycle diary to support personalized symptom tracking in patients diagnosed with premenstrual disorders. By enabling the assessment of menstrual cycle–related symptom fluctuations in real-life contexts, this approach paves the way for future studies to develop interventions aimed at managing premenstrual symptoms and ultimately improving care for patients with premenstrual disorders.

## Supporting information

S1 File Supporting information. (DOCX)
