# Supplementary material for: Study protocol for the development of a digital menstrual cycle diary for routine mental health and gynecological care: A human-centered design approach
Source: PLoS One. 2026 Mar 9;21(3):e0342586. doi: 10.1371/journal.pone.0342586 (PMC12970904; doi:10.1371/journal.pone.0342586)
Supplement: S1 File — (DOCX) [file pone.0342586.s001.docx]

**Supporting information**

**Title manuscript:**

Study protocol for the development of a digital menstrual cycle diary for routine mental health and gynecological care: A human-centered design approach

**Authors:**

Michèle Schmitter^1,2*^, Astrid Cantineau^3^, Marije aan het Rot^4^, Annemiek Lely^5^, Tom Verhage^1^, Michelle N. Servaas^1^ & Harriëtte Riese^1^

^1^ Department of Psychiatry, Groningen, University Medical Center Groningen, University of Groningen, The Netherlands

^2^ Depression Expertise Centre, Pro Persona Mental Health Care, Nijmegen, The Netherlands

^3^ Department of Obstetrics and Gynecology, University Medical Center Groningen, University of Groningen, Groningen, The Netherlands

^4^ Department of Psychology (Clinical), University of Groningen, Groningen, The Netherlands

^5^ Dutch Brain Foundation, The Hague, The Netherlands

**Table of contents**

[**1. Clinical and menstrual cycle information** 2](#_Toc216430831)

[**2. Topic guide for value specification phase** 6](#_Toc216430832)

[**3. Interview guide evaluation phase** 8](#_Toc216430833)

[**4. Evaluation survey for clinicians who did not use the diary in evaluation phase** 13](#_Toc216430834)

# **1. Clinical and menstrual cycle information**

During the screening for the value specification and evaluation phase, patients will report their basic clinical details, menstrual cycle information and tracking methods used. See Table A for the specific questions.

Table A

*Clinical and menstrual cycle information*

| **Question in Dutch (original)** | **English translation** |
| --- | --- |
| 1) Bent u op dit moment onder behandeling bij een medisch specialist voor klachten die gerelateerd zijn aan uw menstruele cyclus?  ○Ja ○Nee  Indien ja, kunt u aangeven bij welk specialisme u onder behandeling bent, waarvoor, en in sinds wanneer? | 1) Are you currently receiving treatment from a medical specialist for symptoms associated with your menstrual cycle?  ○Yes ○No  If yes, please specify the medical specialty, the condition being treated, and the date the treatment began. |
| 2) Bent u in het verleden onder behandeling geweest bij medisch specialist voor klachten die gerelateerd zijn aan uw menstruele cyclus?  ○Ja ○Nee  Indien ja, kunt u aangeven bij welk specialisme u onder behandeling was, waarvoor, en in welke periode? | 2) Have you in the past received treatment from a medical specialist for symptoms associated with your menstrual cycle?  ○Yes ○No  If yes, please specify the medical specialty, the condition being treated, and the period during which the treatment took place. |
| 3) Bent u op dit moment onder behandeling voor een psychiatrische aandoening?  ○Ja ○Nee  Indien ja, voor welke diagnose of klachten bent u onder behandeling, en sinds wanneer? | 3) Are you currently receiving treatment for a psychiatric condition? ○Yes ○No  If yes, specify the diagnosis or symptoms for which you are receiving treatment, and indicate when the treatment commenced. |
| 4) Gebruikt u op dit moment medicatie (bijvoorbeeld antidepressiva, antipsychotica, stemmingsstabilisatoren, genderbevestigende hormonen, hormoonzalf of antihistamine)?  ○Ja ○Nee  Indien ja, waarvoor gebruikt u medicatie en sinds wanneer?  *Indien iemand deze info niet wil delen:*  Denkt u dat deze medicatie van invloed is op uw menstruele cyclus?  ○Ja ○Nee | 4) Are you currently using any medication (for example, antidepressants, antipsychotics, mood stabilizers, gender-affirming hormones, hormone cream, or antihistamines)? ○Yes ○No  If yes, what medication are you using and since when?  *If someone prefers not to share this information:* Do you think this medication affects your menstrual cycle? ○Yes ○No |
| 5) Gebruikt u momenteel anticonceptie?  ○Ja ○Nee  Indien ja, wat voor anticonceptie?  - Anticonceptiepil  - Ander hormonaal (e.g., Prikpil, Implanon, Mirena spiraal)  - Anders, namelijk…  Hoe lang gebruikt u deze anticonceptie al?  Indien nee, heeft u in het verleden anticonceptie gebruikt?  ○Ja ○ Nee  Zo ja, gedurende welke periode, wat voor anticonceptie en hoe lang bent u al zonder anticonceptie? | 5) Are you currently using contraception? ○Yes ○No  If yes, what type of contraception?  - Contraceptive pill  - Other hormonal (e.g., Depo-Provera injection, Implanon, Mirena IUD)  - Other, namely...  How long have you been using this contraception?  If no, have you used contraception in the past? ○Yes ○No  If yes, specify the duration of use, the type of contraception, and any periods during which you were not using contraception. |
| 6) Heeft u momenteel (nog) een menstruele cyclus?  - Ja  - Nee, ik heb geen eierstokken of baarmoeder meer  - Nee, ik ben in de overgang (geweest)  - Nee, ik ben zwanger  - Nee, ik ben recent bevallen/ik geef borstvoeding  - Anders namelijk… | 6) Do you currently (still) have a menstrual cycle?  - Yes  - No, I no longer have ovaries or a uterus  - No, I am in (or have been in) menopause  - No, I am pregnant  - No, I have recently given birth/I am breastfeeding  - Other, namely... |
| 7) Heeft u een regelmatige menstruele cyclus?  ○Ja ○Nee | 7) Do you have a regular menstrual cycle? ○Yes ○No |
| 8) Hoeveel dagen duurt uw menstruele cyclus normaal gesproken? | 8) How many days does your menstrual cycle typically last? |
| 9) Wat was de eerste dag van uw laatste menstruatie? | 9) What was the first day of your last menstruation? |
| 10) Hoe houdt u uw cyclus bij?  - App (bv. Clue, Flo)  - (Digitaal) Agenda  - Op papier  - Anders, namelijk…  - Niet | 10) How do you track your cycle?  - App (e.g., Clue, Flo)  - (Digital) Calendar  - On paper  - Other, namely...  - Not at all |
| 11) Heeft u ooit op advies of instructie van een zorgprofessional uw menstruele cyclus bijgehouden?  ○Ja ○Nee  Zo ja, kunt u beschrijven hoe u dit heeft gedaan en welke hulpmiddelen of methoden u heeft gebruikt (bijv. papieren dagboek, digitale apps, etc.)?  - App (bv. Clue, Flo)  - (Digitaal) Agenda  - Op papier  - Anders, namelijk… | 11) Have you ever monitored your menstrual cycle following the recommendation of a healthcare professional? ○Yes ○No  If yes, describe how you tracked your menstrual cycle and which tools or methods you used (e.g., paper diary, mobile application, wearable device).  - App (e.g., Clue, Flo)  - (Digital) Calendar  - On paper  - Other, namely... |
| 12) Merkt u veranderingen (bv. in uw stemming, energie of slaap) tijdens uw cyclus waardoor het (nog) moeilijker voor u is om dagelijkse dingen te doen, zoals voor uzelf zorgen, werken, of contact hebben met anderen?  ○Ja ○Nee  Zo ja, welke veranderingen en wanneer? | 12) Do you experience changes during your menstrual cycle (e.g., in mood, energy, or sleep) that make it more difficult to carry out daily activities such as self-care, work, or social interactions? ○Yes ○No  If yes, what changes do you notice and when? |
| 13) Heeft u verder nog opmerkingen of aanvullende informatie die u wilt delen? | 13) Do you have any further comments or additional information you would like to share? |

*Note*. These questions are assessed during the screening for focus groups and for the summative evaluation (see manuscript for details). Eligibility was based on questions 1, 2, and 12; patients responding ‘yes’ to at least one were included.

# **2. Topic guide for value specification phase**

The focus group session will start with brief introductions, during which the moderator (MS1), the user experience (UX) designer (TV), and the individual with lived experience (AL) will explain their roles in the project. Participants will then be invited to share their first name and what motivated them to join the focus group. The moderator will provide background information on the purpose of the digital menstrual cycle diary, including a picture of the paper-based PMS calendar diary on the slides, background of the PETRA diary, and how PETRA functions within mental healthcare. She will explain that PETRA is a digital diary tool integrated into the electronic health record, enabling personalized symptom monitoring and automated feedback, and emphasize that participant input is essential the design and functionality of the new menstrual cycle diary. Table B gives the topic guide.

Table B

*Topic guide focus group sessions during value specification phase*

| **Question in Dutch (original)** | **English translation** |
| --- | --- |
| **Achtergrond** | **Background** |
| - Wie van jullie is bekend met het papieren dagboek? | - Who is familiar with the paper diary? |
| - Wie heeft het papieren dagboek ooit bijgehouden? | - Who has ever completed the paper diary? |
| **Thema 1 – Behoeften** | **Theme 1 – Needs** |
| - Wanneer of waarvoor zou je een menstruatiedagboek willen gebruiken? | - When or for what purpose would you want to use a menstrual cycle diary? |
| - Wat wil je leren van het monitoren van de cyclus en klachten? | - What would you want to learn from monitoring the cycle and symptoms? |
| - Wat is voor jou het belangrijkste om te weten na het invullen van het dagboek? | - What is the most important thing for you to know after completing the diary? |
| - Welke informatie en inzichten wil je terugzien in de feedback? | - What information and insights would you want to receive in the feedback? |
| - Wat zou in de feedback moeten staan om diagnostiek en behandelkeuze te ondersteunen? (behandelaren) | - What should be included in the feedback to support diagnosis and treatment decisions? (clinicians) |
| - Hoe denken cliënten hierover? | - What do patients think about this? |
| - Wat is prettig aan het papieren dagboek, en welke functies zou het digitale dagboek ook moeten hebben? | - What do you like about the paper diary, and which of its functions should also be included in the digital diary? |
| - Wat zou een digitaal dagboek beter of minder goed maken dan een papieren dagboek? | - What would make a digital diary better or worse than a paper diary? |
| - Zou je liever een digitaal of papieren dagboek gebruiken? | - Would you prefer to use a digital or paper diary? |
| **Thema 2 – Monitoren** | **Theme 2 – Monitoring** |
| - Welke klachten zou je willen monitoren? | - Which symptoms would you want to monitor? |
| - Welke klachten zouden standaard uitgevraagd moeten worden? | - Which symptoms should always be included as standard items? |
| - Welke informatie over de cyclus zou je willen monitoren? | - What menstrual cycle information would you want to track? |
| - Zou je het dagboek vaker dan één keer per dag willen invullen? | - Would you want to complete the diary more than once per day? |
| - Op welk moment van de dag zou je de informatie willen invoeren? | - At what time of day should the information be entered? |
| **Thema 3 – Feedback** | **Theme 3 – Feedback** |
| - Wat is voor jou het belangrijkste om na het invullen van het dagboek te weten te komen? | - What is the most important thing for you to know after using the diary? |
| - Zou je de feedback willen kunnen delen met naasten of een volgende behandelaar? Met wie en op welke manier? | - Would you want to share the feedback with family or a future clinician? With whom and how? |
| **Afsluiting** | **Closing** |
| - Wat is jullie het meest bijgebleven van dit gesprek? | - What stood out to you most from this discussion? |
| - Is er nog iets dat niet aan bod is gekomen? | - Is there anything we have not addressed yet? |

# **3. Interview guide evaluation phase**

To assess the diary’s usability and impact, we will conduct interviews with clinicians and patients who have recently used the diary. The interview guides for patients and clinicians are provided in Tables C and D, respectively.

Table C

*Semi-structured interview guide for patients in evaluation phase*

| **Questions in Dutch (original)** | **English translation** |
| --- | --- |
| 1. **Kunt u kort vertellen wat voor u de belangrijkste reden was om het digitale menstruatiedagboek te gebruiken?**   Essentiële prompts:   - - Heeft iemand u geadviseerd om het te gebruiken (bijvoorbeeld uw behandelaar)? Zo ja, wie?   - Wat hoopte u dat het dagboek u zou opleveren?   - Had u al eerder iets vergelijkbaars geprobeerd om uw klachten en cyclus bij te houden? Zo ja, hoe heeft u dat gedaan? Wat waren uw ervaringen? | 1. **Can you briefly describe your main reason for using the digital menstrual cycle diary?**   Essential prompts:   - Did anyone advise you to use it (e.g., your clinician)? If yes, who? - What did you hope to gain from using the diary? - Had you tried something similar before to track your symptoms and cycle? If yes, how did you do it? What were your experiences? |
| 1. **Wat waren uw ervaringen met het digitale menstruatiedagboek?**   Essentiële prompts:   - - Vond u het dagboek makkelijk in gebruik? Zo ja, wat maakte het gebruiksvriendelijk?   - Heeft u uitdagingen ervaren bij het gebruik? Voorbeeld van iets dat lastig was of niet werkte?   - Heeft u technische problemen gehad? Zo ja, welke?   - Was de feedback goed te begrijpen? Zo nee, wat was moeilijk?   - Hoe hebt u de samenwerking met uw behandelaar ervaren bij het gebruik van het dagboek? Was het dagboek samen opgesteld en besproken?   - *Indien eerder gebruikt:* vergeleken met eerdere methoden, wat vond u beter of slechter aan het digitale menstruatiedagboek? | 1. **What were your experiences using the digital menstrual cycle diary?**   Essential prompts:   - Did you find the diary easy to use? If yes, what made it user-friendly? - Did you face challenges while using it? Can you give an example of something that was difficult or did not work? - Did you encounter technical issues? If yes, which ones? - Was the feedback easy to understand? If not, which aspects were difficult? - How did you experience working with your clinician while using the diary? Was the diary set up and feedback discussed together? - *If previously used:* Compared to previous tracking methods, what did you find better or worse about the digital menstrual cycle diary? |
| 1. **Wat heeft het gebruik van het digitale menstruatiedagboek u opgeleverd?**   Essentiële prompts:   - - Heeft het dagboek inzicht gegeven in de relatie tussen klachten en menstruele cyclus? Hoe?   - Heeft het dagboek input geleverd voor de behandeling? Voorbeeld van invloed op behandelkeuzes?   - Heeft het dagboek bijgedragen aan de relatie met de behandelaar? Hoe merkte u dat? | 1. **What did you gain from using the digital menstrual cycle diary?**   Essential prompts:   - Did the diary provide insight into the relationship between your symptoms and menstrual cycle? How? - Did the diary inform your treatment? Can you give an example of how it influenced treatment decisions? - Did the diary contribute to your relationship with your clinician? How did you notice this? |
| 1. **Stel u zou het digitale menstruatiedagboek mogen aanpassen of verbeteren – wat zou u veranderen?**   Essentiële prompts:   - - Welke onderdelen van de instellingen (bv. anticonceptie, gemiddelde cyclusduur, startdatum laatste menstruatie) vond u nuttig of overbodig? Waarom?   - Welke onderdelen van de feedback waren nuttig of overbodig?   - Zou u verbeteringen willen zien in inhoud, vormgeving of gebruiksgemak? Zo ja, welke en hoe zou dit het gebruik verbeteren?   - Zijn er zaken die u gemist heeft?   - Zou u het dagboek aanraden? | 1. **If you could modify or improve the digital menstrual cycle diary, what would you change?**   Essential prompts:   - Which settings (e.g., contraception, average cycle length, start date of last menstruation) were useful or unnecessary? Why? - Which parts of the feedback were useful or unnecessary? - Would you like improvements in content, design, or usability? If yes, which ones, and how would they improve use? - Were there things you felt were missing? - Would you recommend the diary? |
| 1. **Zijn er nog andere zaken die wij nog niet hebben besproken, maar volgens u wel van belang zijn?** | 1. **Are there any other topics we have not discussed that you think are important?** |

Table D

*Semi-structured interview guide for clinicians in evaluation phase*

| **Questions in Dutch (original)** | **English translation** |
| --- | --- |
| 1. **Kunt u kort vertellen wat voor u de belangrijkste reden was om het digitale menstruatiedagboek te gebruiken met uw cliënte?**   Essentiële prompts:   - Wat maakte dat u besloot het dagboek in te zetten? - Wat hoopte u dat het uw cliënten of uzelf zou opleveren? - Had u al eerder gewerkt met menstruatiedagboeken (bijvoorbeeld op papier of digitaal)? - Zo ja, kunt u uitleggen welke dagboeken dat waren? Wat waren uw ervaringen met die dagboeken? | 1. **Can you briefly describe your main reason for using the digital menstrual cycle diary with your client?**   Essential prompts:   - What made you decide to use the diary? - What did you hope it would achieve for your clients or for yourself? - Have you used menstrual diaries before (for example, on paper or digitally)? - If so, which diaries were they, and what were your experiences with them? |
| 1. **Wat waren uw ervaringen met het digitale menstruatiedagboek?**   Essentiële prompts:   - Vond u het dagboek makkelijk in gebruik? Zo ja, kunt u toelichten wat het gebruiksvriendelijk maakte? - Heeft u uitdagingen ervaren met gebruik van het dagboek? Kunt u een voorbeeld geven van iets wat u lastig vond aan het dagboek of niet werkte? - Heeft u technische problemen gehad? Zo ja, welke? - Was de feedback goed te begrijpen? Indien nee, wat waren aspecten die u moeilijk vond? - Was het opstarten en bespreken van het dagboek goed in te passen in het behandelcontact? Indien nee, waarom niet? - *Indien eerder gebruikt:* Als u het digitale menstruatie dagboek vergelijkt met dagboeken die u eerder heeft ingezet, zijn er dan aspecten aan dit dagboek die u beter of juist minder goed vond? Zo ja, welke? | 1. **What were your experiences using the digital menstrual cycle diary?** Essential prompts:  - Did you find the diary easy to use? If so, can you explain what made it user-friendly? - Did you experience any challenges when using the diary? Can you give an example of something you found difficult or that didn’t work well? - Did you encounter any technical problems? If so, what were they? - Was the feedback easy to understand? If not, which aspects did you find difficult? - Was starting and discussing the diary easy to fit into your treatment sessions? If not, why not? - *If other diaries were used before:* Compared to diaries you previously used, are there aspects of the digital menstrual cycle diary that you found better or worse? If so, which ones? |
| \| 1. **Wat heeft het gebruik van het digitale menstruatiedagboek u opgeleverd?**   Essentiële prompts:   - Heeft het dagboek u geholpen inzicht te krijgen in de samenhang tussen klachten en de menstruele cyclus? Kunt u toelichten op welke manier dat inzicht ontstond? - Heeft het dagboek input geleverd voor de behandeling? Kunt u een voorbeeld geven van hoe het dagboek de behandeling beïnvloedde? Heeft het dagboek ook invloed gehad op uw behandelkeuzes? Zo ja, kunt u hier een voorbeeld van geven? - Heeft het dagboek bijgedragen aan de relatie met uw cliënte? Hoe merkte u dat in het contact met uw cliënte? \| \| --- \| | 1. **What did you gain from using the digital menstrual cycle diary?**   Essential prompts:   - Has the diary helped you gain insight into the relationship between symptoms and the menstrual cycle? Can you explain how this insight arose? - Has the diary provided input for treatment? Can you give an example of how it influenced the treatment? Has it also affected your treatment decisions? If so, can you give an example? - Has the diary contributed to your relationship with your client? How did you notice this in your interactions with your client? |
| 1. **Stel u zou het digitale menstruatiedagboek mogen aanpassen of verbeteren – wat zou u veranderen?**   Essentiële prompts:   - Welke onderdelen uit de instellingen (bv. anticonceptie gebruik, gemiddelde cyclus duur, laatste menstruatie) vond u nuttig of juist overbodig en waarom? - Welke onderdelen uit de feedback vond u nuttig of juist overbodig? - Zou u verbeteringen willen zien op het gebied van inhoud, vormgeving of gebruiksgemak? Zo ja, welke? In hoeverre denkt u dat deze verbetering het gebruik makkelijker of effectiever zou maken? - Zijn er zaken die u gemist heeft in het gebruik van het dagboek? - In het algemeen, zou u het dagboek nogmaals inzetten? | 1. **If you could modify or improve the digital menstrual cycle diary, what would you change?**   Essential prompts:   - Which elements from the settings (e.g., contraceptive use, average cycle length, last menstruation) did you find useful or unnecessary, and why? - Which elements from the feedback did you find useful or unnecessary? - Would you like to see improvements in terms of content, design, or usability? If so, which ones? To what extent do you think these improvements would make the diary easier or more effective to use? - Are there any aspects you felt were missing in the use of the diary? - In general, would you use the diary again? |
| 1. **Zijn er nog andere zaken die wij nog niet hebben besproken, maar volgens u wel van belang zijn?** | 1. **Are there any other topics we have not discussed that you think are important?** |

# **4. Evaluation survey for clinicians who did not use the diary in evaluation phase**

In the evaluation phase, we will administer an anonymous survey to clinicians to identify reasons for not using the digital menstrual cycle diary. The survey questions are presented in Table E.

Table E

*Survey questions for not using the digital menstrual cycle diary*

| **Questions in Dutch (original)** | **English translation** |
| --- | --- |
| **Heeft u het** **digitale menstruatiedagboek tot nu toe ingezet bij patiënten?**  ○ Ja ○ Nee | **Have you used the digital menstrual cycle diary with patients so far?**  ○ Yes ○ No |
| **Wat zijn voor u redenen geweest om het digitale menstruatiedagboek niet in te zetten?**  ☐ Ik kende het PETRA dagboek niet  ☐ Ik wist niet goed wanneer het relevant is  ☐ Ik wist niet hoe het werkt  ☐ Het kost teveel tijd  ☐ Het past niet in mijn werkwijze  ☐ Ik heb twijfels over de meerwaarde  ☐ Anders, namelijk: | **What have been your reasons for not using the digital menstrual cycle diary?**  ☐ I was not familiar with the PETRA diary  ☐ I was unsure when it is relevant  ☐ I did not know how it works  ☐ It takes too much time  ☐ It does not fit with my way of working  ☐ I have doubts about its added value  ☐ Other, namely: |
| **Wat zou u nodig hebben om het digitale menstruatiedagboek wel te gebruiken?**  ☐ Korte instructie/handleiding  ☐ Voorbeelden van geschikte casussen  ☐ Inbedding in bestaande processen (bijv. standaard bij intake)  ☐ Positieve ervaringen van collega’s  ☐ Onderbouwing van de klinische meerwaarde  ☐ Anders, namelijk: | **What would you need in order to use the digital menstrual cycle diary?**  ☐ Short instruction/manual  ☐ Examples of suitable cases  ☐ Integration into existing processes (e.g., standard at intake)  ☐ Positive experiences from colleagues  ☐ Evidence of clinical added value  ☐ Other, namely: |
| **In welke discipline werkt u?**  ☐ Psychiater  ☐ Psycholoog/GZ/KP-psycholoog  ☐ Verpleegkundig specialist  ☐ Gynaecoloog  ☐ AIOS/ANIOS  ☐ Anders: | **Which discipline do you work in?**  ☐ Psychiatrist  ☐ Psychologist/GZ/KP Psychologist  ☐ Nurse Specialist  ☐ Gynecologist  ☐ Resident/Junior Doctor (AIOS/ANIOS)  ☐ Other: |
